# Changes in isometric mid-thigh pull peak force and symmetry across anterior cruciate ligament reconstruction rehabilitation phases

**DOI:** 10.3389/fresc.2024.1418270

**Published:** 2024-07-05

**Authors:** Johannes P. J. Stofberg, Kerith Aginsky, Mariaan van Aswegen, Mark Kramer

**Affiliations:** ^1^ Centre for Health and Human Performance (CHHP), North-West University, Potchefstroom, South Africa; ^2^ Ribstein Center for Sports Medicine and Research, Wingate Institute, Netanya, Israel; ^3^ Physical Activity, Sport, and Recreation (PhASRec) Research Focus Area, North-West University, Potchefstroom, South Africa

**Keywords:** ACL, bilateral, kinematics, kinetics, unilateral

## Abstract

**Background:**

Whether functionally relevant strength assessments, such as the isometric mid-thigh pull (IMTP), can be used either bilaterally or unilaterally to evaluate and guide rehabilitation progress in those with anterior cruciate ligament reconstruction (ACLR) is under-researched. This study assessed changes in peak force (PF) and asymmetry across 3 phases for bilateral and unilateral IMTP assessments in patients with ACLR. Peak isometric force from the IMTP was compared to peak torque from isokinetic dynamometry as well as against a cohort of healthy, uninjured individuals.

**Method:**

Participants (ACLR, *n* = 15) completed bilateral and unilateral IMTP assessments at weeks 12 (baseline), 16 (phase 3), and 20 (phase 4) of rehabilitation to evaluate changes in PF and asymmetry. Asymmetry was evaluated using the asymmetry angle. Isometric data from the IMTP were compared to that from an isokinetic dynamometer as well as against a cohort of healthy, uninjured participants (*n* = 63) allowing for a detailed analysis of limb-specific force production.

**Results:**

The PF during the bilateral IMTP increased for both the injured (0.94 N/kg) and uninjured (0.26 N/kg) limbs from baseline to phase 4, whereas the PF of the injured limb increased by 1.5 N/kg during the unilateral IMTP in the same time frame. Asymmetry values systematically reduced by ∼1% and ∼0.5% for the bilateral and unilateral IMTP tests from baseline to phase 4. Significant differences in PF of the injured limb were evident between those with ACLR and healthy controls across all phases (*p* = 0.022–0.001). The rate of progression in PF capacity was dependent on test type, amounting to 0.1 and 0.2 body weights per week for the bilateral and unilateral IMTP respectively. Small-to-large correlations (*r* = 0.12–0.88) were evident between IMTP PF and peak torque from the isokinetic dynamometer as well as between asymmetry metrics from both tests.

**Conclusion:**

The findings suggest that IMTP PF has potential for monitoring changes in PF and asymmetry during the ACLR rehabilitation progress. Both injured limb and uninjured limb show improvement in force-generating capacity, implying a positive adaptation to rehabilitation protocols. The findings highlight that ACLR is a unilateral injury that requires bilateral rehabilitation.

## 1 Introduction

Athletes and active individuals aiming to regain optimal knee function and return to pre-injury levels of activity often undergo anterior cruciate ligament reconstruction (ACLR) (1, 2). However, the journey to full recovery extends beyond surgical intervention, encompassing comprehensive rehabilitation processes that focus on restoring strength, stability, and confidence in the affected limb (3). A crucial component in the recovery trajectory is the evaluation of lower extremity isometric strength which provides valuable insights into the neuromuscular control and functional capacity of the knee joint post-reconstruction (2, 4).

Conventionally, isokinetic dynamometry is used to quantify the recovery of muscle strength across various joints such as the knee, specifically the quadriceps and hamstrings, thereby providing a quantitative basis to inform return-to-play (RTP) decisions (5). Specifically, this method assesses muscle performance to ensure athletes meet established criteria for strength and symmetry before progressing through the rehabilitation stages towards full sport participation. While an isokinetic evaluation remains a key part of the decision-making process, recent suggestions advocate for more functionally relevant assessments based on the assumption that such assessments can offer insights that are more applicable to real-world performances, including the ability to differentiate between the capabilities of injured and uninjured limbs that tend to coincide with on-field performance outcomes (6, 7).

The isometric mid-thigh pull (IMTP) test is, at least to some extent, a functionally relevant strength test used to determine the maximal force generation capability of athletes in a more controlled setting (8). The IMTP is considered a reliable and valid measure of maximal strength, with high levels of reliability and validity when tested correctly (9, 10). The static nature of the IMTP, along with its ease of administration, offers safety and lower risk of injury compared to dynamic maximal strength tests like the one-repetition maximum (1RM) tests, which pose concerns about technique and handling heavy weights (11). The IMTP has been linked to improved performance in dynamic activities such as powerlifting, weightlifting, and sprinting (12–16), and its relevance in multi-directional field sports like rugby league and union is evident (17, 18). Additionally, its strong associations with sprint performance and change-of-direction ability highlight its value, suggesting that the IMTP is an effective tool for assessing changes in strength and the associations with an athlete's explosive power and sprinting ability (15). Whether the IMTP can be used as a potential monitoring tool during ACLR rehabilitation process has not been previously investigated, nor the extent to which peak forces differ between injured and healthy groups. Therefore, the extent to which such findings map onto more functionally compromised groups, such as those with ACLR, requires further exploration.

Evidence-based decision-making, which rests on the use of quantifiable data such as strength assessments, limb symmetry, balance capabilities, as well as perceptions of pain and readiness, is an essential component in ACLR rehabilitation (19). More specifically, objective criteria are typically used to ensure patients meet specific criteria before advancing through each rehabilitation phase (20). Such methods aim to prevent premature return to sport, associated with high reinjury rates by using an objective, evidence-based approach (21). Despite such approaches, ACL reinjury rates have not significantly declined over the past two decades (16), prompting a re-examination of current rehabilitation strategies and whether key aspects can be improved. Possible factors for the lack of change in ACL incidences likely include the use of inappropriate tests, insufficient test sensitivity, misinterpretation of results by clinicians, or increased physical demands in sports, heightening reinjury risk. This indicates the need for a nuanced approach in rehabilitation and return-to-sport processes, reflecting the evolving nature of ACL injury mechanisms and the possible limitations of some current testing methodologies. The use of objective data provide a framework for clinicians to make informed decisions about rehabilitation phase timing and progression, ensuring decisions are based on concrete performance outcomes (22, 23).

While clinicians widely adopt evidence-based criteria [e.g., limb symmetry index > 90% for strength and hop tests] in making RTP decisions, patients may still face challenges in fully restoring muscle strength, neuromuscular control, movement quality, and psychological readiness upon returning to sports. These factors are identified by Kyritsis et al. (24) as critical in injury risk mitigation, and given the individual variability in rates of tissue healing and response to ACL Rehabilitation (ACLR), the necessity for personalized rehabilitation approaches to more effectively restore muscle strength, coordination, and symmetry, must be underscored (25). Additionally, assessment should include the functioning of the uninvolved limb since inattention of the uninjured limb can worsen individual differences, leading to muscle imbalances and compensatory mechanisms that may increase the risk of concomitant injury (26), and suboptimal recovery (27). Therefore, a balanced approach that addresses both limbs is crucial for effective recovery and injury prevention, considering the range of individual healing and response patterns. To date, no study has investigated the use of the bilateral and/or unilateral IMTP in the ACLR population across multiple phases and its potential benefits in the decision-making process. This lack of research emphasizes the need for a detailed study to explore how effectively the IMTP can differentiate strength capacities between the injured limb and the uninjured limb.

Subsequently, the primary objectives of this study were to: (i) assess the efficacy of IMTP in distinguishing between the injured limb and the uninjured limb throughout various rehabilitation phases, (ii) evaluate changes in the magnitude of the bilateral and unilateral peak force (PF) values across the rehabilitation phases, (iii) compared changes in asymmetry of the limbs across the different phases, (iv) compare PF of an ACLR cohort to healthy individuals, and (v) correlate PF from the IMTP to those from an isometric test using an isokinetic dynamometer. The study focused on tracking changes in PF across these rehabilitation stages where we hypothesized that IMTP would effectively differentiate between the injured limb and both the uninjured limb and control group, offering insights into strength variations during recovery.

## 2 Materials and methods

### 2.1 Participants

A minimum sample size of 15 participants was calculated *a priori* using the following input parameters: (i) moderate effect size (f) of 0.25; (ii) type-1 error rate of 5%; (iii) type-2 error rate of 20%; (iv) 9 repeated measures; and (v) an expected correlation of 0.50 among repeated measures. Although a total of 18 ACLR participants volunteered for the study, the final cohort retained for analysis consisted of 15 individuals who had undergone ACLR (ACLR group; *n* = 3 omitted due to incomplete data). The IMTP performances from the ACLR group were compared to that of a control group (*n* = 63) consisting of healthy individuals without injury. The inclusion criteria for the ACLR group were individuals aged 14–30 who had undergone ACLR and were assessed between 12 and 20 weeks post-operatively. The control group was matched for age, sex, and activity level whereby both groups consisted of active athletes (rugby, hockey, netball). Exclusion criteria for both groups included any additional or recent (within the last 6 months) lower limb surgeries or conditions that could affect strength measurement.

All participants completed an informed consent form prior to data collection and voluntarily enrolled in the research study. This study was approved by the Health Research Ethics Committee of the Faculty of Health Sciences of the institution (ethics number: NWU-00335-21-A1) and was conducted according to the ethical guidelines and principles of Ethics in Health Research: Principles, Processes and Structures (28) and other international ethical guidelines applicable to this study.

### 2.2 Experimental procedures

Due to the high levels of effort required for both isokinetic and IMTP testing, the assessment of the IMTP was only initiated in week 12 of rehabilitation. The primary justifications include that (i) there is considerable variability in tendon graft healing rates whereby the cellular proliferation phase begins ∼3–6 months post-operatively (29), and the graft is potentially at its weakest during weeks 4–8 (30). Testing was repeated every 4 weeks as this is a typical timeline for reasonable anatomical adaptation to occur within a training/rehabilitation mesocycle and falls within the recommendations for phase progressions in those with ACLR (30, 31).

#### 2.2.1 Isometric mid-thigh pull

**Figure 1:**
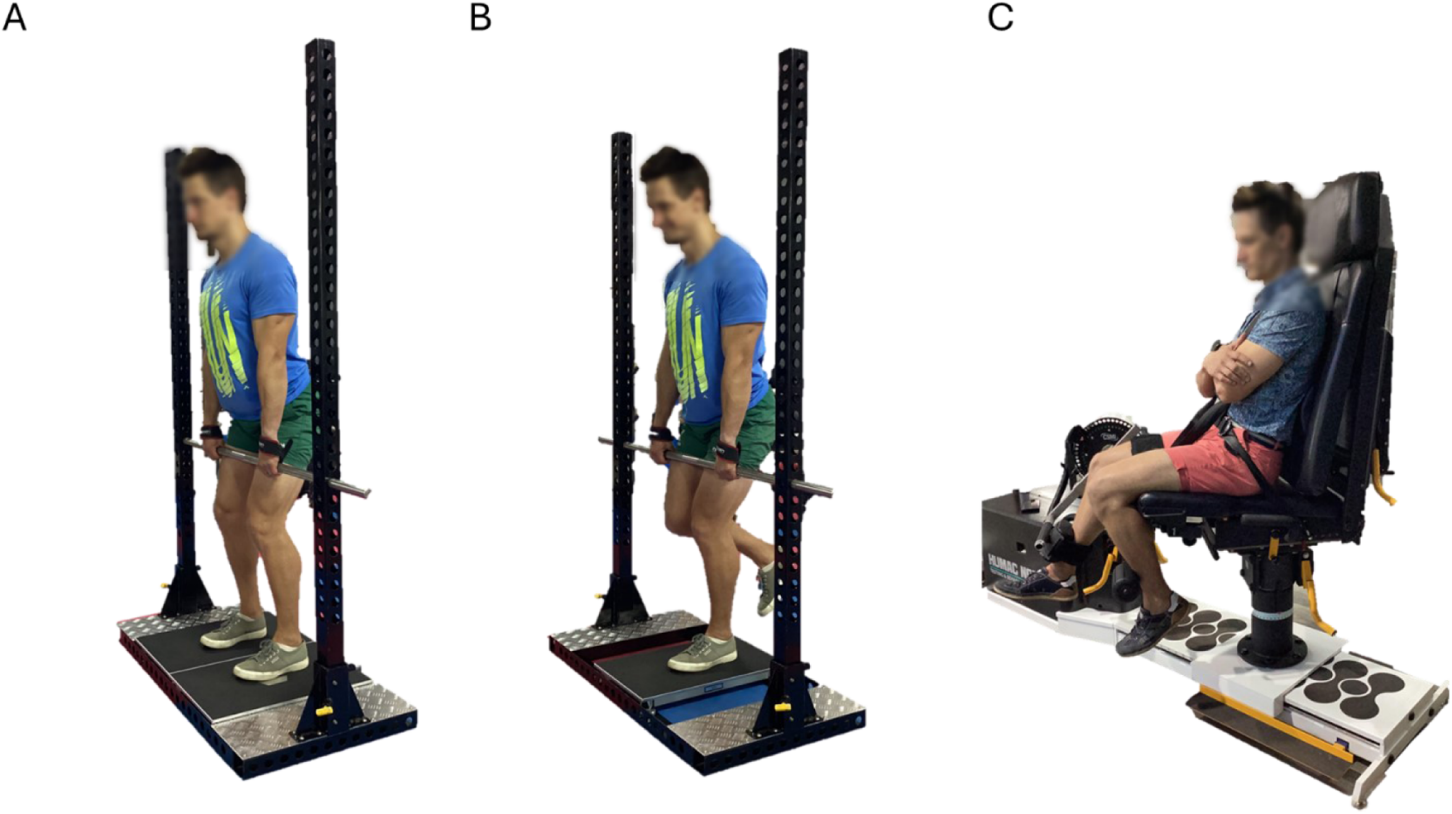
Experimental setup for the isometric mid-thigh pull (IMTP) and isokinetic dynamometry. (Panel **A**): bilateral IMTP; (panel **B**): unilateral IMPT; and (panel **C**): isokinetic dynamometry.

All IMTP assessments were conducted on a Hawkin Dynamics force plate system (3rd Generation, model 0486; Westbrook, Maine, USA) with a sampling frequency of 1,000 Hz (Figure 1). The force plates used show excellent validity (intercept: 0.01 CI 95% [−0.03, 0.05]; slope: 0.98, CI95% [0.93, 1.04) across all parameters of interest (32). Peak force values were normalized to bodyweight (BW), where BW was measured on the force plates for a 1 s interval immediately before the initiation of the IMTP test.

The IMTP assessment protocol, as described by Comfort et al. (33) was used for the present study. Briefly, before the assessment, each participant underwent a warm-up routine including general and specific components. The general warm-up included 10 min of cycle ergometry at light-to-moderate intensity, rated 9–13 on the Borg scale (ACSM, 2013:145–146) followed by BW squats and lunges. The dynamic warm-up phase involved low-load and moderate-load mid-thigh pulls, comprising three sets of 3 s IMTP trials with a 60 s rest interval between sets, at 50%, 75% and 90% of their perceived maximum effort.

After the warm-up, participants stepped onto a dual force plate system and adjusted to ensure no slack in the bar, preventing pre-tensioning of the muscles (Figures 1A,B). The BW was recorded using the system, during which participants were instructed to maintain a still position for 1 s. Then, participants were informed of the test commencement, followed by a 3 s countdown. They were prompted to “exert maximum force into the ground as swiftly and forcefully as possible” for 4 s, with verbal encouragement provided.

The IMTP was performed with participants in a posture similar to the second pull position of the clean, with knee angles of 125°–145° and hip angles of 140–150° (33). To avoid grip strength limiting maximal force production, a clean grip supplemented with lifting straps and hands secured to the bar was used. Grip width was individually measured and documented for standardization and reproducibility. Foot positioning (neutral, inverted, or everted) was measured and recorded for consistency across trials. After each trial, participants remained stationary on the system until force exertion was recorded. A minimum of three successful trials were conducted, with a 2 min rest interval between trials, and the ensemble average data were retained for analysis. All trials were completed bilaterally and unilaterally (Figures 1A,B).

The IMTP was conducted at weeks 12, 16, and 20 for the ACLR group to track progression through rehabilitation. The control group underwent a single assessment to establish baseline strength values such that values from the ACLR group could be compared across different time points.

#### 2.2.2 Isokinetic dynamometry

Isometric testing of the isolated quadriceps muscle group was performed on an isokinetic dynamometer (Cybex II, HUMAC®/Norm™; Computer Sports Medicine, Inc., Stoughton, MA, USA) with a sampling frequency of 100 Hz (Figure 1C).

Isometric testing of the quadriceps muscle group was performed on the Humac NORM isokinetic dynamometer. The reliability of the Humac NORM isokinetic dynamometer was evaluated to be strong [intraclass correlation coefficient (ICC) = 0.74–0.89] (34, 35). Participants completed a warm-up on a cycle ergometer, during which they were informed of the testing procedure and that the test should be at maximal pain-free effort. The warm-up, including five to 12 BW squats and lunges within a functional range of motion (ROM), was explained.

The participant was positioned and stabilized in the seated position with the chair 85° reclined and thighs supported by the seat. This allowed a testing ROM from 75°–90° of flexion to maximal knee extension (36). Stabilization belts were used to stabilize the tested leg, thigh, and thorax. Participants were instructed to cross their arms across their chest. The lateral femoral epicondyle was aligned with the mechanical rotation axis of the dynamometer's lever arm. During isometric testing, the resistance pad was positioned proximally to negate anterior shear force to the ACL. Range of motion (ROM) was set by taking the knee through full extension and flexion. ROM was adjusted for those unable to attain full extension and flexion and re-assessed with subsequent testing and increased throughout the rehabilitation phases where possible. A gravity torque correction was performed.

Isometric testing was conducted at 60° of knee flexion to minimize anterior shear force to the ACL. A specific warm-up was completed by each participant that included two repetitions each of knee extension at 25%, 50%, 75%, and a single repetition at 100% effort for 5 s. This helped warm up the muscles and familiarize participants with the machine. Participants were monitored for pain or discomfort during the warm-up. After the specific warm-up and a 1 min rest period, isometric knee extension was tested with a 5 s active contraction followed by a 10 s rest period for five repetitions. The ensemble average of all valid repetitions were retained for analysis whereby the raw data were exported to Matlab for processing.

The order of testing (i.e., IMTP vs. isokinetics) was not randomized as the order of testing was not anticipated to be a confounding factor.

#### 2.2.3 Asymmetry

Given that the goal of the rehabilitation of ACLR patients is to return to comparable between-leg performances, both in terms of strength and movement coordination, an evaluation of the asymmetry is pertinent. Although several different asymmetry calculations exist, arguably the most robust is the asymmetry angle (AA) (37). Within the context of the present study, asymmetry was calculated as follows:

$$\mathrm{AA}\;(\text{\%})=\frac{\left(45^{\circ }-\;\tan^{-1}\left[\frac{\mathrm{injured}}{\mathrm{uninjured}}\right]\right)}{90^{\circ }}\cdot 100$$

### 2.3 Data processing and analysis

Raw data for the isokinetic- and IMTP tests were imported to Matlab (version R2021b, The MathWorks, MA, USA) for processing. For the isokinetic data, the torque-time waveforms were ensemble averaged across all repetitions such that the PF from the mean curve could be extracted for analysis. For the IMTP, the force-time waveforms were smoothed using a fourth-order, zero-lag Butterworth filter with cutoff frequencies of 8 Hz. All IMTP repetitions were ensemble averaged where the maximum force recorded during the 5 s interval was reported as PF which was also normalized to body mass.

### 2.4 Statistical analyses

All analyses were completed using JASP (JASP Team, version 0.18.1, Netherlands) and the R programming language (R Core Team, version 2022.04.01, RStudio, Posit Software PBC, URL: https://posit.co/download/rstudio-desktop/). Data were evaluated for normality using the Shapiro-Wilk test where deviations from normality were accepted at *p* < 0.05. A repeated measures ANOVA (rmANOVA) was used from the *afex* package to contrast: (i) PF data of the involved and uninvolved limbs across three phases of rehabilitation. (ii) unilateral and bilateral differences in PF across the rehabilitation phases, and (iii) differences in the bilateral and unilateral AA across the rehabilitation phases (38). Paired contrasts were evaluated using the *emmeans* package where Cohen's d was used as a measure of the standardized effect size. The magnitude of the Cohen's d coefficient was qualitatively interpreted as: trivial:<0.20; small: 0.20–0.59; moderate: 0.60–1.19; large: 1.20–2.00; very large: >2.00. For all repeated measures analyses the sphericity assumption was evaluated using Mauchly's test of sphericity and the effect size reported as generalized eta squared (*η*_g_^2^). For instances where sphericity was violated the Greenhouse-Geisser correction was implemented using the *afex* package.

A regression analysis was conducted using the *ggstatsplot* package to evaluate the relationship between changes in peak force (dependent variable) and the phase of rehabilitation, where the phase was captured as the specific week in which testing took place (independent variable). Finally, a Spearman Rank correlation analysis was conducted using the *correlation* package to evaluate the associations between PF from the IMTP and the peak torque from an isokinetic dynamometer during an isometric test. The magnitude of the correlation coefficient was qualitatively interpreted as follows: negligible: 0.00–0.10 =; weak: 0.10–0.39; moderate: 0.40–0.69; strong: 0.70–0.89; very strong: 0.90–1.00 (39). In all instances where multiple comparisons conducted (e.g., repeated measures ANOVA, correlation), *p*-values were adjusted using the Holm correction to minimize the type-1 error rate.

## 3 Results

The relevant descriptive and inferential statistics for the between-group anthropometric data are presented in Table 1. No statistically significant between-group differences were evident for the anthropometric parameters measured.

**Table 1:** Descriptive and inferential summary statistics for between-group anthropometric data.

Variable	ACLR Group	Healthy Control	*p*-value
Gender (M/F)	10/4	54/9	
Age (years)	22.47 ± 2.45	22.10 ± 2.07	0.565
Height (m)	1.77 ± 0.80	1.84 ± 0.79	0.766
Body mass (kg)	85.94 ± 18.13	93.21 ± 11.31	0.058

**Figure 2:**
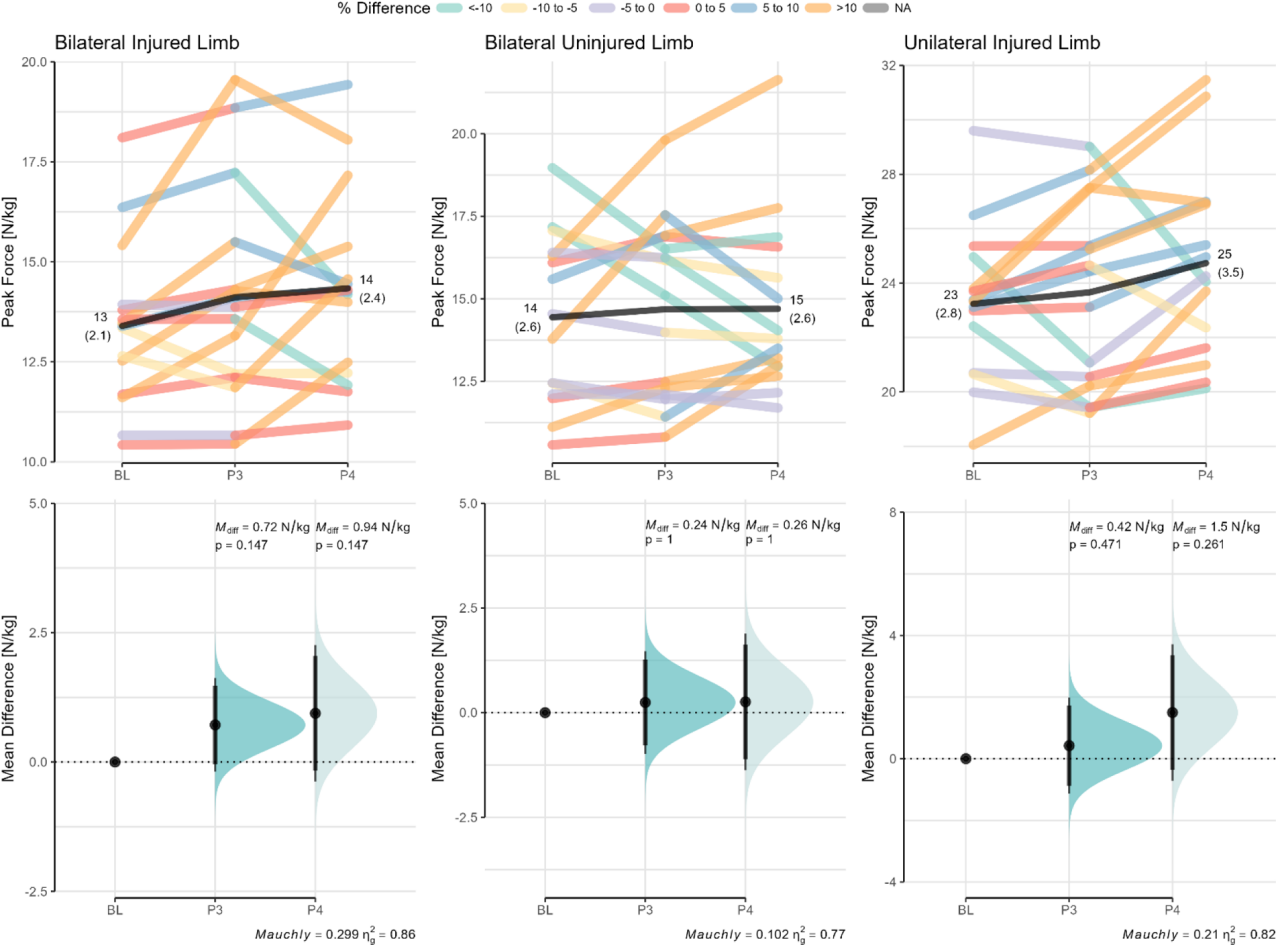
Changes in bilateral and unilateral peak forces across the rehabilitation phases for the injured and uninjured limbs. Paired data are shown for each participant across the different phases. Mean differences (M_diff_) relative to baseline (BL) with the CI95% are shown in the bottom panels. The paired values are color-coded based on the percentage difference relative to baseline. The *p*-value of Mauchly's test of sphericity and generalized eta squared (*η*_g_^2^) are reported in the caption of the bottom panels. M_diff_, mean difference; BL, baseline (or phase 2); PF, peak force; P3, phase 3; P4, phase 4.

The relative peak forces (PF; N/kg) for both the injured and uninjured limbs during the bilateral IMTP test as well as the injured limb during the unilateral IMTP test across all phases are shown in Figure 2 (top panels). The mean differences (M_diff_) in PF of each subsequent phase are shown relative to the baseline measures (Figure 2, bottom panels). It is evident from the data that both the injured and uninjured limbs are progressively improving as evidenced by the shift in mean PF although it is pertinent to mention that there is considerable variability in the ability to generate the PF across the rehabilitation phases.

**Figure 3:**
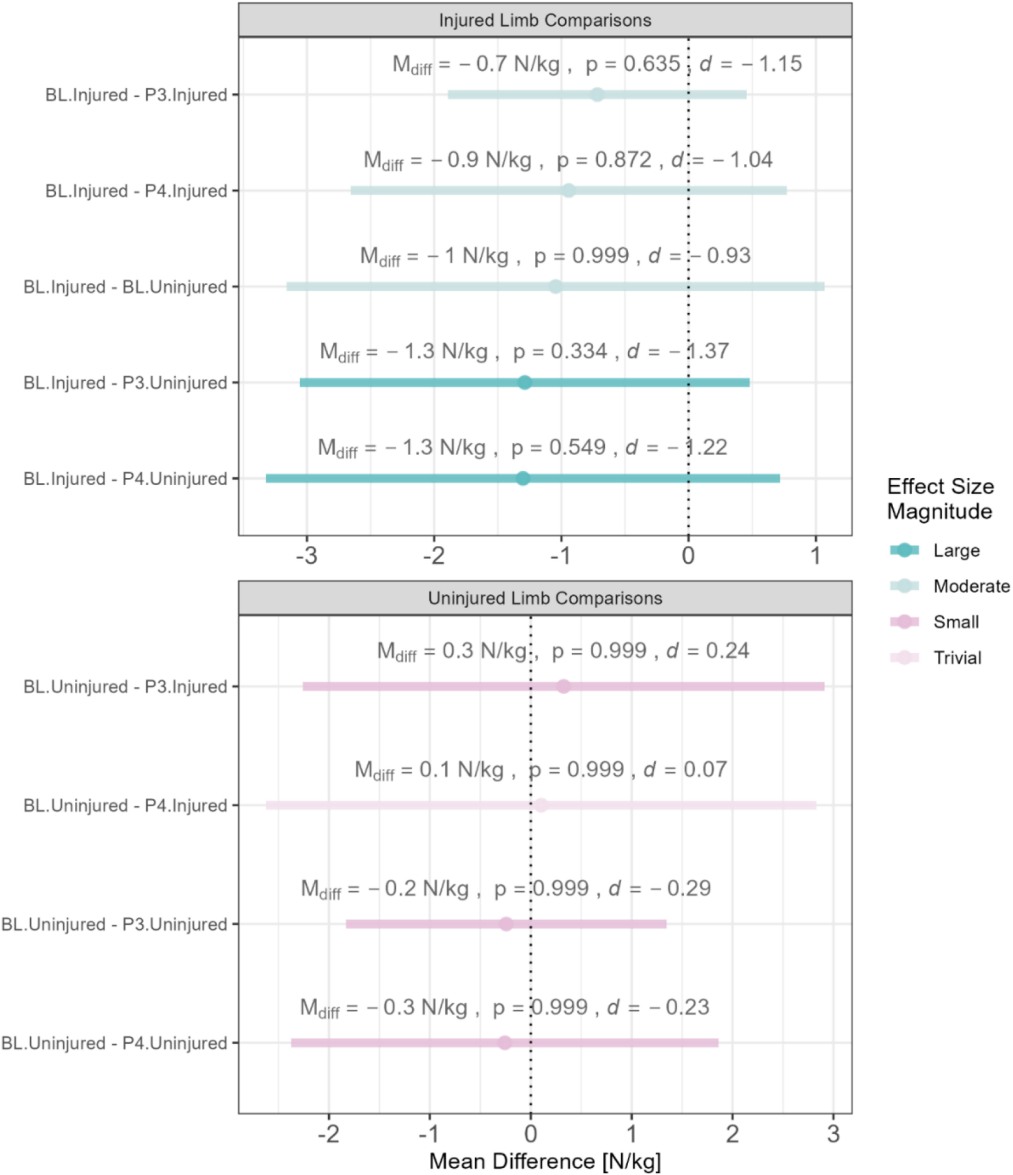
Between-limb comparison of relative peak forces across all phases. BL, baseline; P3, phase 3; P4, phase 4 L; M_diff_, mean difference; N, newtons; d, Cohen's d; mean differences are color-coded based on the magnitude of the standaridized effect size.

A contrast of the relative PF between the limbs across all phases is provided in Figure 3. Although the between-limb differences were not significantly different, it is important to note the magnitude of the standardized effect sizes which range from trivial to large.

**Figure 4:**
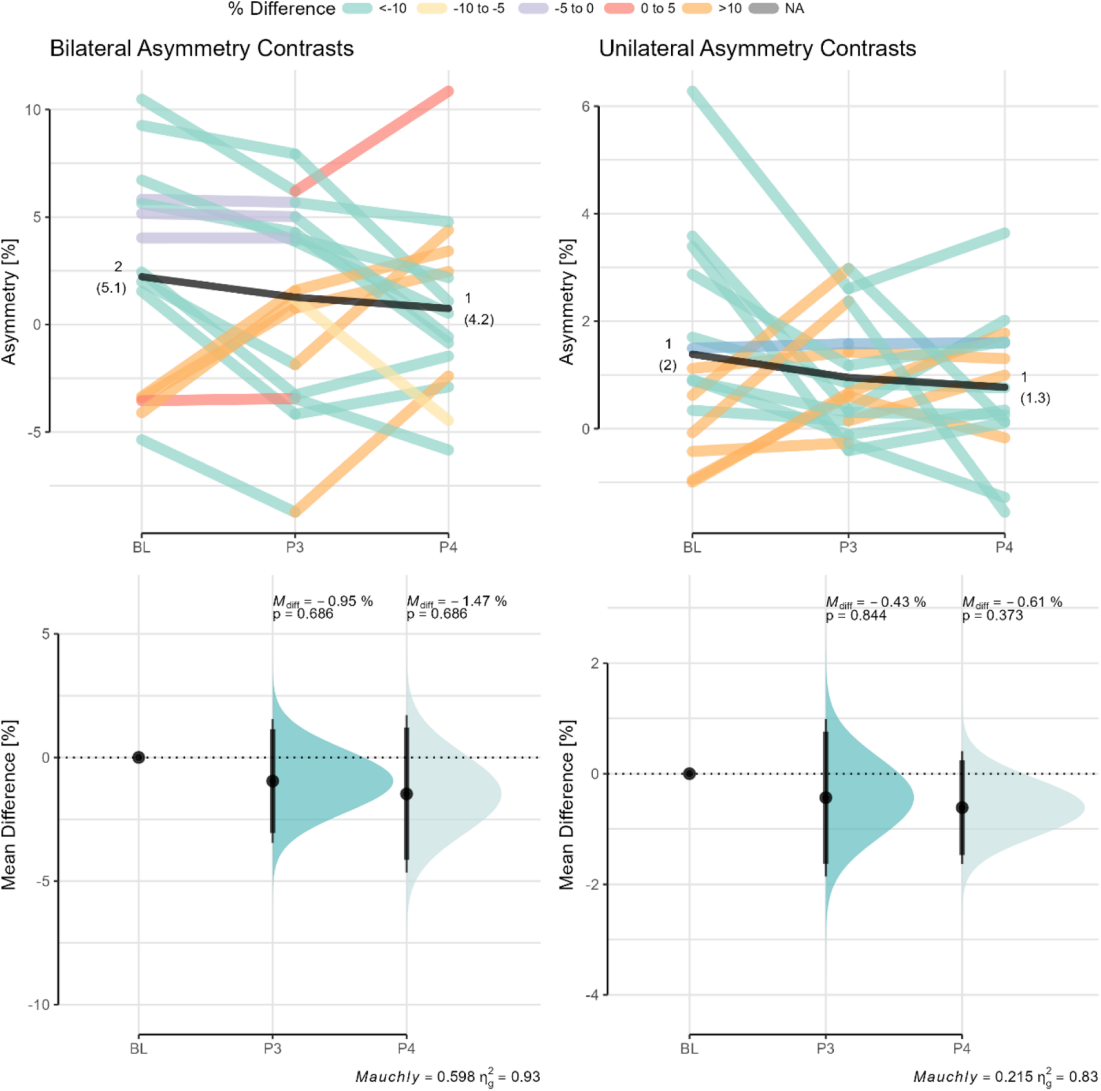
Changes in bilateral and unilateral asymmetry angles across phases. Paired data are shown for each participant across the different phases with data points color coded based on the percentage difference relative to baseline. Mean differences (M_diff_) with the CI95% relative to baseline are shown in the bottom panel. The *p*-value of Mauchly's test of sphericity and generalized eta squared (*η*_g_^2^) are reported in the caption of the bottom panels BL, baseline (or phase 2); P3, phase 3; P4, phase 4.

The progression of the bilateral and unilateral AA across rehabilitation phases are highlighted in Figure 4. Positive asymmetry values indicate that the uninjured limb yielded higher values compared to the injured limb for the metric of interest, whereas negative asymmetry values indicate the opposite. Substantially variability is evident between phases with fairly large percentage changes from baseline especially for the injured limb.

**Figure 5:**
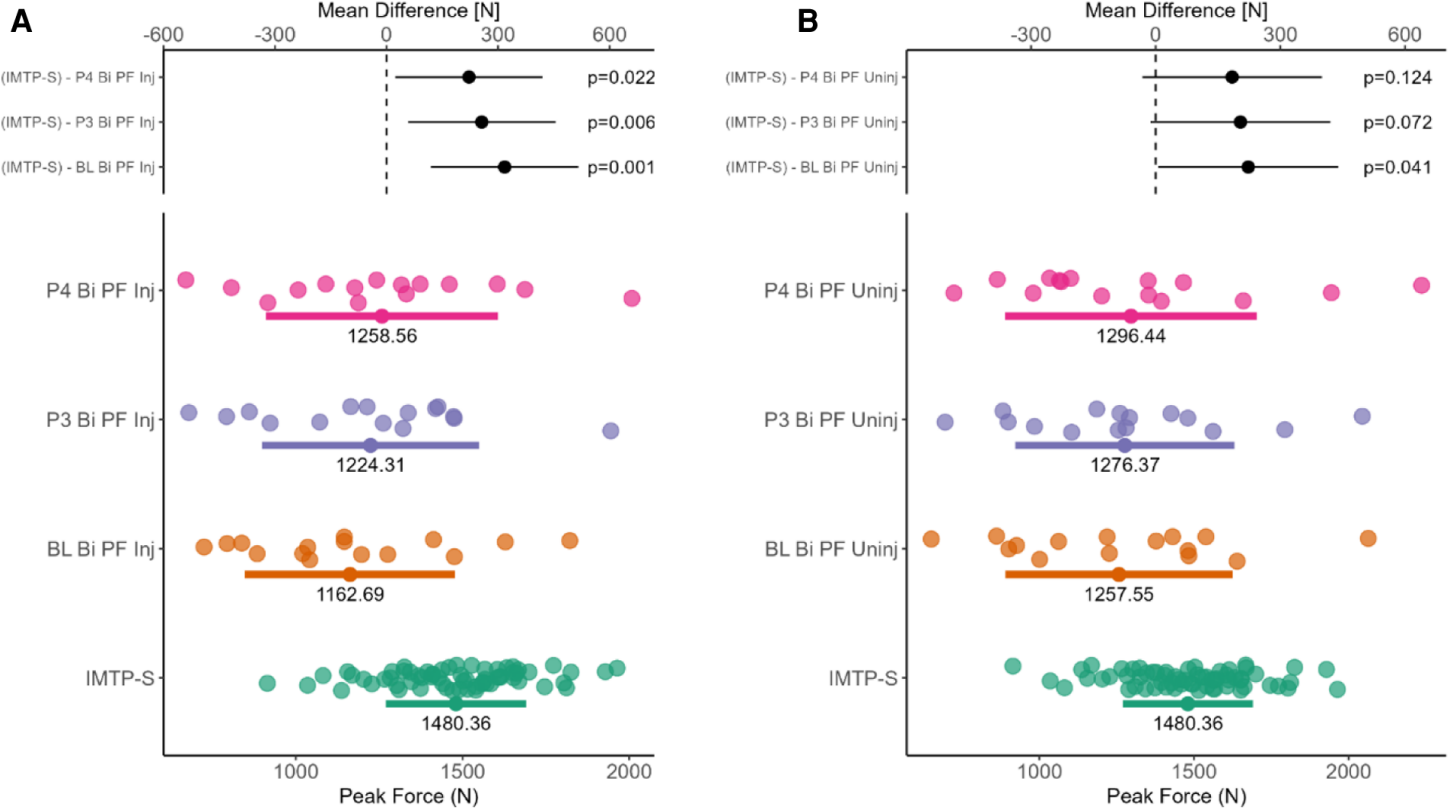
Mean between-group differences for bilateral IMTP peak force. (Panel **A**) shows mean difference in peak force between the strong limb of healthy individuals and the injured limb of the ACLR group across three phases. (Panel **B**) shows mean difference in peak force between the strong limb of healthy individuals and the uninjured limb of the ACLR group across three phases.

The PF values of the injured and uninjured limbs across the different phases of the ACLR group were compared to the strongest limb of the control group (see Figure 5). Significant between-group differences are evident for PF of the injured limb across all rehabilitation phases (see Figure 5A). Relative to the control group, the uninjured limb of the ACLR group showed significant difference only at baseline but not across phases 3–4 (see Figure 5B).

**Figure 6:**
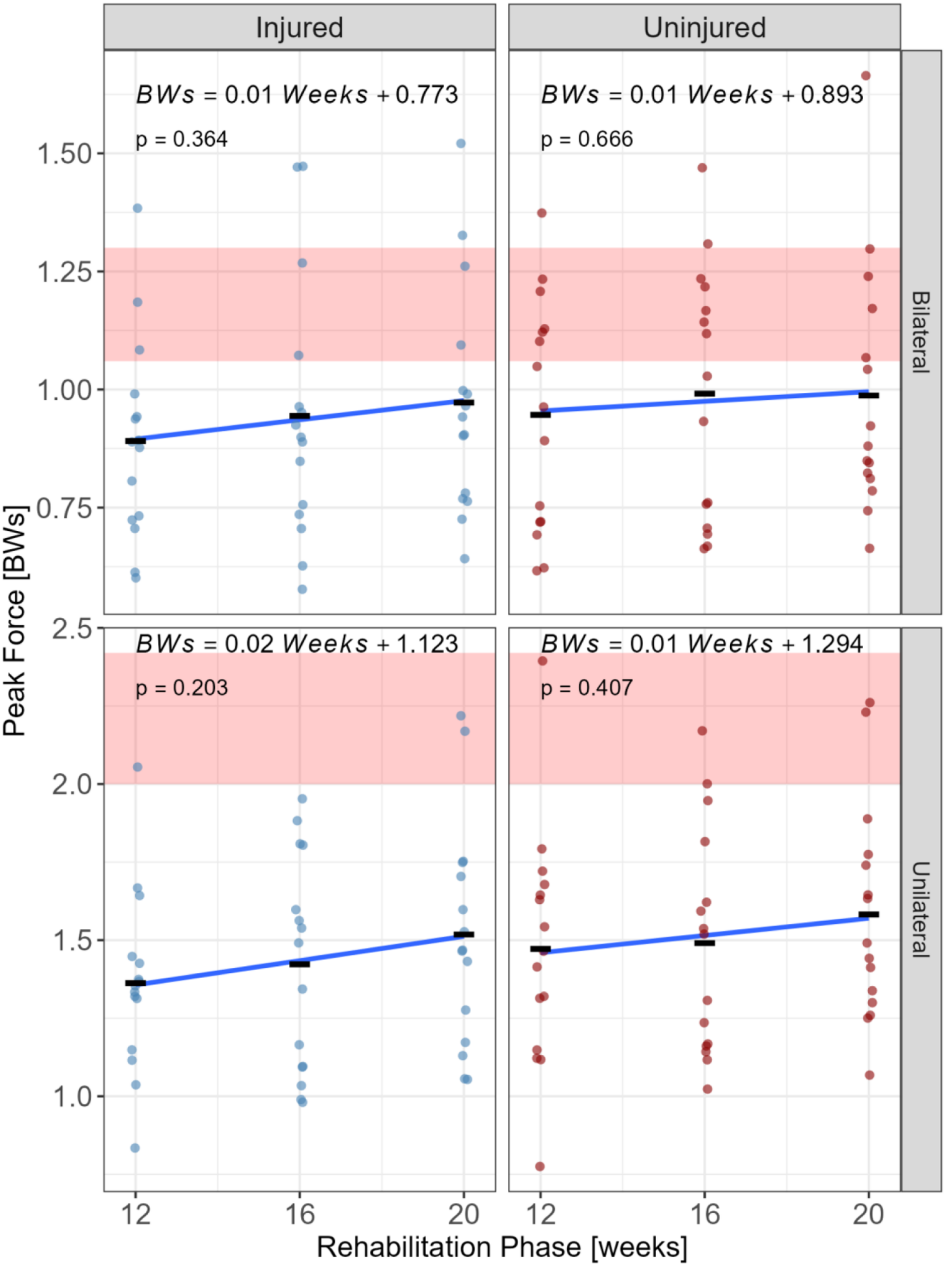
Scatter plot illustrating the progression of peak force (PF) normalized to body weights (BWs) in the injured and uninjured limbs during the rehabilitation phase (weeks 12, 16, and 20). The data are split according to the test type (bilateral and unilateral). The linear regression lines (blue) depict the rate of improvement over time. The red shaded region indicates the PF ranges of uninjured healthy individuals.

The rate of improvement in the relative PF across the phases of both the injured and uninjured limbs of the ACLR group is shown in Figure 6 where the data are split based according to the test type (i.e., bilateral vs. unilateral). It is clear that throughout the rehabilitation phases from weeks 12, 16, and 20, both limbs demonstrate a small, but consistent improvements in relative PF. The rate of improvement appears to be dependent on the test type and would suggest that the ACLR group would require a minimum of approximately 28 weeks to achieve the minimum relative PF threshold of healthy individuals.

**Figure 7:**
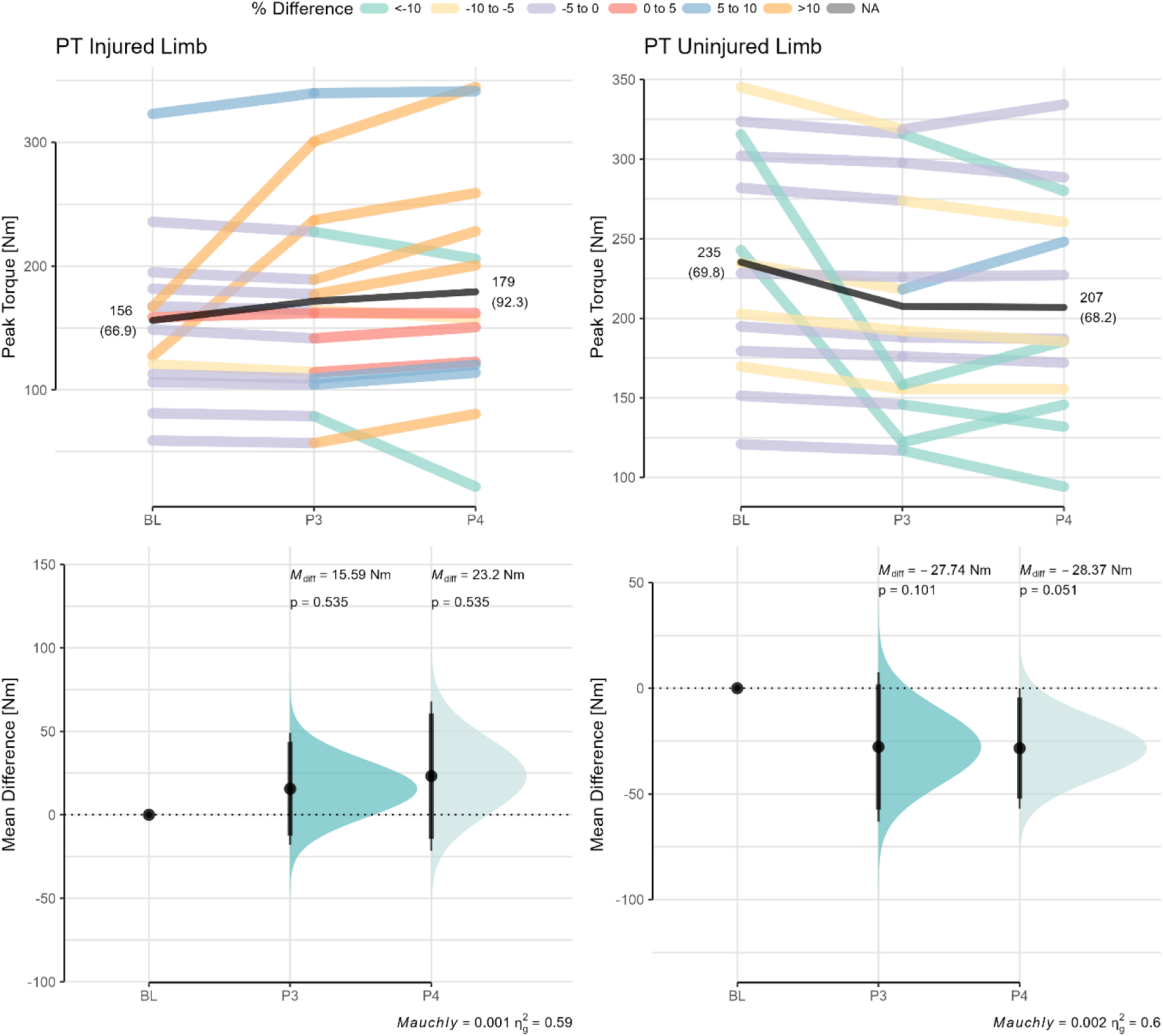
Isometric peak torque values from an isokinetic dynamometer as a function of rehabilitation phase. Paired data are shown for each participant across the different phases with data points color coded based on the percentage difference relative to baseline. Mean differences (M_diff_) with the CI95% relative to baseline are shown in the bottom panel. The *p*-value of Mauchly's test of sphericity and generalized eta squared (*η*_g_^2^) are reported in the caption of the bottom panels BL, baseline (or phase 2); P3, phase 3; P4, phase 4.

For reference purposes the peak torque values from the isokinetic dynamometer across the rehabilitation phases are highlighted in Figure 7.

**Figure 8:**
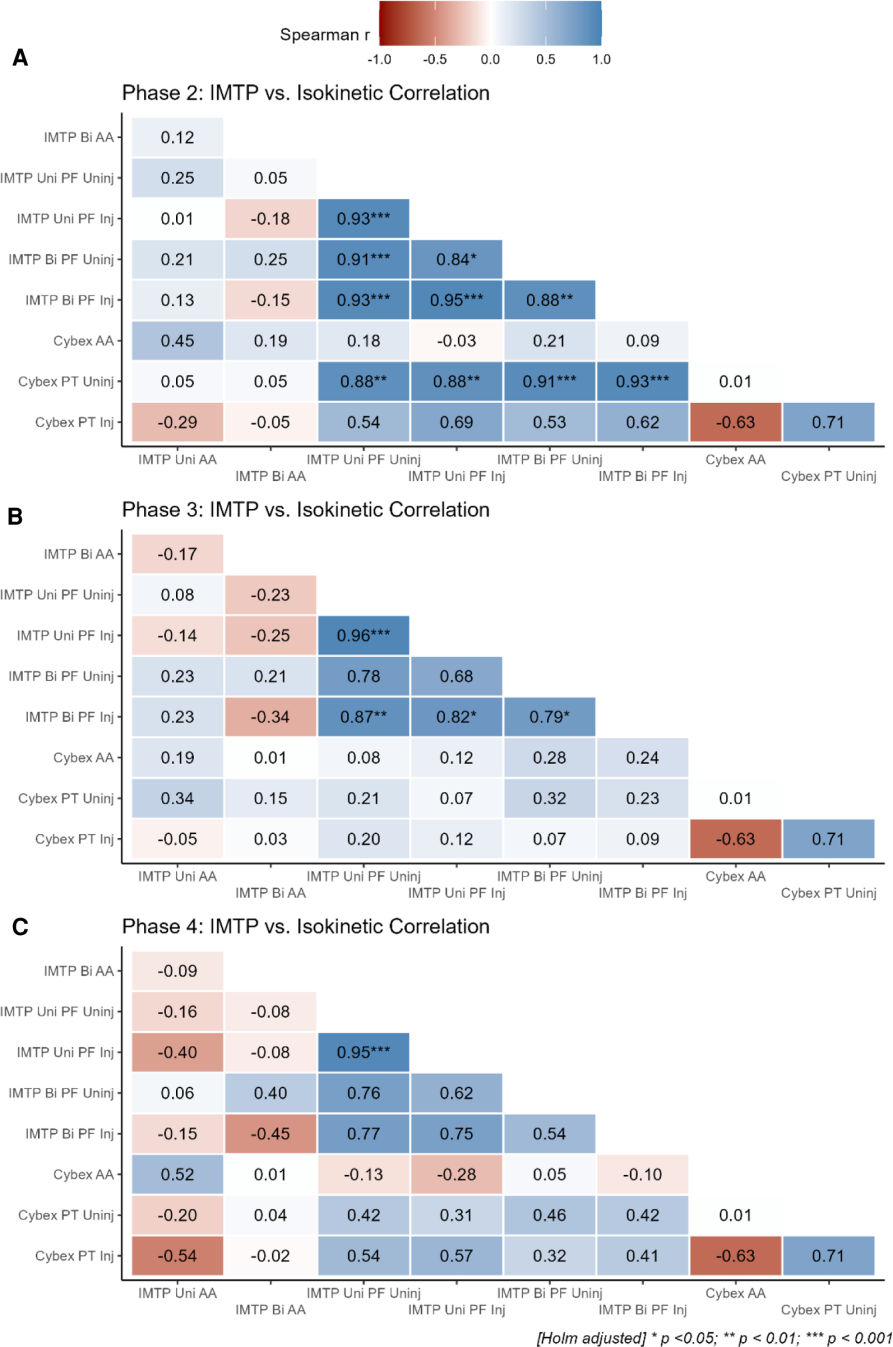
Correlation analysis between metrics from the IMTP test and isokinetic dynamometry across the different phases. Panel **A**: IMTP vs. isokinetic correlations for phase 2; Panel **B**: IMTP vs. isokinetic correlations for phase 3; Panel **C**: IMTP vs. isokinetic correlations for phase 4. IMTP, isometric mid-thigh pull; Bi, bilateral; Uni, unilateral; AA, asymmetry angle; PF, peak force (N/kg); PT, peak torque; Inj, injured limb; uninj, uninjured limb.

The results of the correlation analysis between PF and asymmetry values from both the IMTP and isokinetic dynamometry (i.e., Cybex) are highlighted in Figure 8. Moderate-to-strong correlations were evident between the PT and PF values of the injured and uninjured limbs depending on the rehabilitation phase. Interestingly, only the AA from the isokinetic dynamometer and unilateral IMTP were moderately associated but no other AA values seem to share any association.

## 4 Discussion

This study evaluated the utility of the IMTP for appraising changes in bilateral and unilateral PF values across multiple phases of rehabilitation. Our research contributes novel insights into the application of IMTP within the sample of ACLR patients assessed by showing: (i) small but meaningful changes in PF capacity across the rehabilitation phases with substantial within-subject variability, (ii) meaningful between-leg differences were present especially regarding the injured limb (*d* = 0.89–1.39), (iii) small changes in asymmetry subjected to substantial within- and between-participant variability, (iv) a mean rate of improvement in relative PF of approximately 0.1 BW's per week, and (v) weak-to-strong correlations between PF and PT capacities, and weak-to-moderate associations between AA across isometric tests. The results tend to support previous research on the importance of incorporating more functional strength assessments in the clearance criteria of ACLR patients (40).

It is well understood that the IMTP is a worthwhile option for practitioners to use in the context of athletic profiling, yet its use within a rehabilitation context, especially for ACLR, has been largely under-researched (8). Following ACLR, the musculoskeletal system undergoes several changes in muscle size, strength, power that ultimately impact the biomechanical and functional outcomes of the individual (41). The ability of practitioners to examine and evaluate the integrity of the musculoskeletal system to guide clinical decision-making is therefore vital, although access to the necessary tools is often limited. It is here that the present study evaluated the use of the IMTP as a potential assessment tool. As an assessment tool, the IMTP has excellent reliability for evaluating PF and relative PF (ICC = 0.99; CV% = 2.5), with the ability to detect a smallest worthwhile change of 112.2 N or 1.3 N/kg (10). Our results would indicate that although improvements in the relative PF were observable across the phases, the IMTP would lack the sensitivity to discriminate between the injured or uninjured limbs on the basis that the injured limb is not necessarily the weaker limb (42). Although differences in both PF and AA were evident between the phases, the within- and between-subject variability evident in the present study would suggest that limb injury status does not necessarily have a bearing on the performance outcome. The latter interpretations are echoed by our findings on two fronts whereby (i) considerable overlap was evident in PF between injured and healthy participants despite statistically significant differences (see Figure 5), and (ii) the AA showed high levels of symmetry between limbs and phases which altered in favor of the injured and uninjured limbs at different time points (see Figure 4). Normative reference values for the AA are presently not available for those with ACLR, therefore whether a quasi risk-threshold exists between healthy and compromised individuals would require further research. Importantly, these findings were similar for the isometric test on the isokinetic dynamometer where marginal improvements in peak torque (PT) were evident between the phases but were not significantly different from baseline. Of interest however, was the finding that the isokinetic dynamometer revealed a progressive weakening of the quadriceps musculature of the uninjured limb relative to baseline which was not evident from the IMTP highlighting the difference between joint isolation compared to a more compound alternative.

It is important to highlight that despite the lack of statistically significant differences between limbs or phases, moderate-to-large standardized differences (*d* = 0.93–1.37) were observed in the sample of participants. The rates of improvement of participants in the current study suggest that the injured limb would require a minimum of approximately 28 weeks to achieve the relative PF values that approximate those of healthy individuals (43). It is worth noting that the rate of improvement appears to be dependent on the test type (i.e., bilateral vs. unilateral), whereby the current set of participants were below the norm for unilateral compared to bilateral IMTP strength (10, 44). It could therefore be inferred that although both bilateral and unilateral strength should be areas of focus, the latter would require more attention. To achieve the requisite strength adaptations across time, a multi-systems approach would necessitate careful planning, possibly by incorporating a linear periodization design such that systematic and logical progressions can be incorporated and monitored as a function of time (31, 41).

During the early phases of ACL rehabilitation, the strength of the quadriceps are objectively evaluated isometrically using an isokinetic dynamometer where it is then also possible to evaluate the magnitude of asymmetry between limbs (45–47). The isokinetically-derived isometric strength values across phases in our study were on par with those of Czaplicki et al. (48) and Karanikas et al. (49) and thus corresponded with the anticipated rates of improvement in quadriceps strength. The IMTP shows potential promise as a tool for evaluating whole-body isometric strength although its use in the monitoring of ACLR patients has not been previously investigated. Our results showed phase-dependent correlations between isometric peak torque and isometric PF which transitioned from small to strong associations from baseline to phase 4 (see Figure 8). Similarly, the associations between AA were trivial-to-moderate between the isokinetic dynamometer and the unilateral IMTP test (*r* = 0.45) and weak for the bilateral IMTP test (*r* = 0.12). It is likely that, due to the differences in the setup between the IMTP (a closed-chain movement) and the isokinetic dynamometer (an open-chain movement), distinct effects on muscle activation and knee joint biomechanics are elicited. More specifically, the IMTP requires a knee angle of ∼40^o^ flexion during the pull which stresses the quadriceps differently compared to the isokinetic dynamometer where an angle of 60^o^ is typically used (34, 50). Whether the IMTP would therefore need to be modified for the evaluation of ACLR patients, such as testing at different knee angles, would however require further research. Moreover, an important distinction between the two tests is that the isokinetic dynamometer isolates a specific joint whereas the IMTP is a multi-joint test. It is therefore also possible to mask the potential inadequacies of the knee joint musculature during an IMTP which might account for some of the variability in test performance observed in the current study. This latter point was highlighted by the finding that the uninjured limb showed continued improvements in IMTP-derived isometric strength (M_diff_ =  + 0.26 *N*/kg), but isometric torque decrements in the isokinetic test (M_diff_ = −28.37 Nm). Intriguingly there is greater within-subject variability during the bilateral compared to unilateral IMTP which does not appear to appreciably subside across phase progressions. Whether different neuromuscular control or compensation strategies are at play would thus require further research.

These results underscore the importance of isometric strength assessments in tracking rehabilitation progress, suggesting that IMTP evaluations, alongside specific isometric tests such as isokinetic dynamometry can provide comprehensive insights into muscle strength recovery. The differences in associations between the injured and uninjured limbs highlight the need for rehabilitation strategies that address not just muscle strength but also neuromuscular control and symmetry between limbs. Our results would appear to suggest that modifications to the IMTP may be necessary to more systematically evaluate those with ACLR and is unlikely to replace, at least in its current form, existing methods of isometric evaluation. Furthermore, our study highlights the complex and variable nature of muscle recovery in ACLR patients, with IMTP offering insights into force generation capabilities throughout rehabilitation phases that appear to coincide with changes in isokinetically-derived PT values, at least for the injured limb. Although IMTP shows promise in monitoring strength recovery, its full potential in capturing all aspects of rehabilitation outcomes needs more exploration and would likely need to be combined with more dynamic evaluations (e.g., jumping). Future research should overcome the limitations inherent in this study by including larger sample sizes, extending the duration of rehabilitation phases, and incorporating more functional recovery measures such as jumping and running tasks. Investigating targeted interventions for limb asymmetry and the strength gap between ACLR patients and healthy individuals could provide insights into optimizing rehabilitation protocols.

Incorporating IMTP assessments into rehabilitation could serve as a benchmark for physical recovery and act as psychological reassurance for patients in their RTP journey, with future studies potentially exploring the correlation between IMTP metrics and psychological readiness as well as with more dynamic tasks such as jumping. This comprehensive approach emphasizes the need for detailed assessment methods to enhance recovery processes and outcomes, supporting a nuanced approach to recovery assessment and intervention in the ACLR population.

## 5 Conclusion

The isometric testing of patients that have undergone ACLR is an important aspect of the rehabilitation journey. Although a call for more functionally relevant tests is reasonable, our study showed that alternatives such as the IMTP, at least within the current context, might not yield clinically informative results when used in isolation. However, it is important to note that both the IMTP and isokinetic dynamometry yielded similar results for the injured limb, but differences for the uninjured limb. Additionally, strength discrepancies between limbs are not regulated by the injury status of that limb as evidenced by the lack of significant between-limb differences which was true for both isokinetic dynamometry as well as the IMTP.

## Data Availability

The datasets presented in this study can be found in online repositories. The names of the repository/repositories and accession number(s) can be found below: Harvard Dataverse (link: https://doi.org/10.7910/DVN/SC6EDC).
